# Hepatitis C Virus Translation Preferentially Depends on Active RNA Replication

**DOI:** 10.1371/journal.pone.0043600

**Published:** 2012-08-24

**Authors:** Helene Minyi Liu, Hideki Aizaki, Keigo Machida, J.-H. James Ou, Michael M. C. Lai

**Affiliations:** 1 Department of Molecular Microbiology and Immunology, Keck School of Medicine, University of Southern California, Los Angeles, California, United States of America; 2 Institute of Molecular Biology, Academia Sinica, Nankang, Taipei, Taiwan; University of California, Merced, United States of America

## Abstract

Hepatitis C virus (HCV) RNA initiates its replication on a detergent-resistant membrane structure derived from the endoplasmic reticulum (ER) in the HCV replicon cells. By performing a pulse-chase study of BrU-labeled HCV RNA, we found that the newly-synthesized HCV RNA traveled along the anterograde-membrane traffic and moved away from the ER. Presumably, the RNA moved to the site of translation or virion assembly in the later steps of viral life cycle. In this study, we further addressed how HCV RNA translation was regulated by HCV RNA trafficking. When the movement of HCV RNA from the site of RNA synthesis to the Golgi complex was blocked by nocodazole, an inhibitor of ER-Golgi transport, HCV protein translation was surprisingly enhanced, suggesting that the translation of viral proteins occurred near the site of RNA synthesis. We also found that the translation of HCV proteins was dependent on active RNA synthesis: inhibition of viral RNA synthesis by an NS5B inhibitor resulted in decreased HCV viral protein synthesis even when the total amount of intracellular HCV RNA remained unchanged. Furthermore, the translation activity of the replication-defective HCV replicons or viral RNA with an NS5B mutation was greatly reduced as compared to that of the corresponding wildtype RNA. By performing live cell labeling of newly synthesized HCV RNA and proteins, we further showed that the newly synthesized HCV proteins colocalized with the newly synthesized viral RNA, suggesting that HCV RNA replication and protein translation take place at or near the same site. Our findings together indicate that the translation of HCV RNA is coupled to RNA replication and that the both processes may occur at the same subcellular membrane compartments, which we term the replicasome.

## Introduction

Hepatitis C virus (HCV) is a positive-sense RNA virus that is estimated to chronically infect as many as 3% of the world's population. As a member of Flaviviridae, HCV is an enveloped virus with a single, positive-stranded RNA around 9.6 kb in length [1]. The viral genome encodes a large viral polyprotein, which is proteolytically processed by cellular signal peptidases and viral proteases into structural (C, E1, E2, and p7) and non-structural (NS2, NS3, NS4A, NS4B, NS5A and NS5B) proteins [2]. Membrane association of the viral proteins is essential for HCV replication, at both steps of RNA transcription and translation [3]–[5]. To decipher the mechanisms by which HCV navigates these steps may necessitate an understanding of the cell biological processes as diverse as cytoplasmic organelle structure and membrane biogenesis and trafficking in the secretory pathway.

Using the HCV subgenomic replicon system as well as infectious virus system, many host factors have been identified to be involved in HCV RNA replication, including the human homologue of the 33-kDa vesicle-associated membrane protein-associated protein (hVAP-33) [6], Golgi-specific brefeldin A resistant guanine nucleotide exchange factor 1(GBF1) [7], Endocytic Rab proteins [8], polypyrimidine-tract-binding protein (PTB) [9], [10], La autoantigen [10], SYNCRIP [11], and host geranylgeranylated proteins and fatty acids [12]. These host proteins that are identified to be in the HCV RNA replication complexes are important in either membrane sorting and trafficking or RNA binding and processing. Some of these host factors, such as PTB and La autoantigen, have been found to regulate HCV translation as well_ENREF_13 by virtue of their binding to the 5′ or 3′ UTR of HCV RNA [13]–[15]. The identification of host proteins with dual-functions in regulating both translation and transcription implies the possibility of coupled transcription/translation of HCV RNA.

The balance between viral RNA transcription and translation is critical for the replication of positive-stranded RNA viruses, since the same RNA is used both for translation and as the template for negative-strand RNA synthesis. Transcription of poliovirus has been reported to be dependent on the translational activity of the viral RNA [16]. On the other hand, the translation of Sindbis virus and vesicular stomatitis virus has been reported to be transcriptionally dependent [17]. Such coupling of transcription-translation has been well documented to confer advantage in maintaining the stability of the RNA molecule in bacteria [18], [19] and also to respond to regulatory signals coordinately.

**Figure 1:**
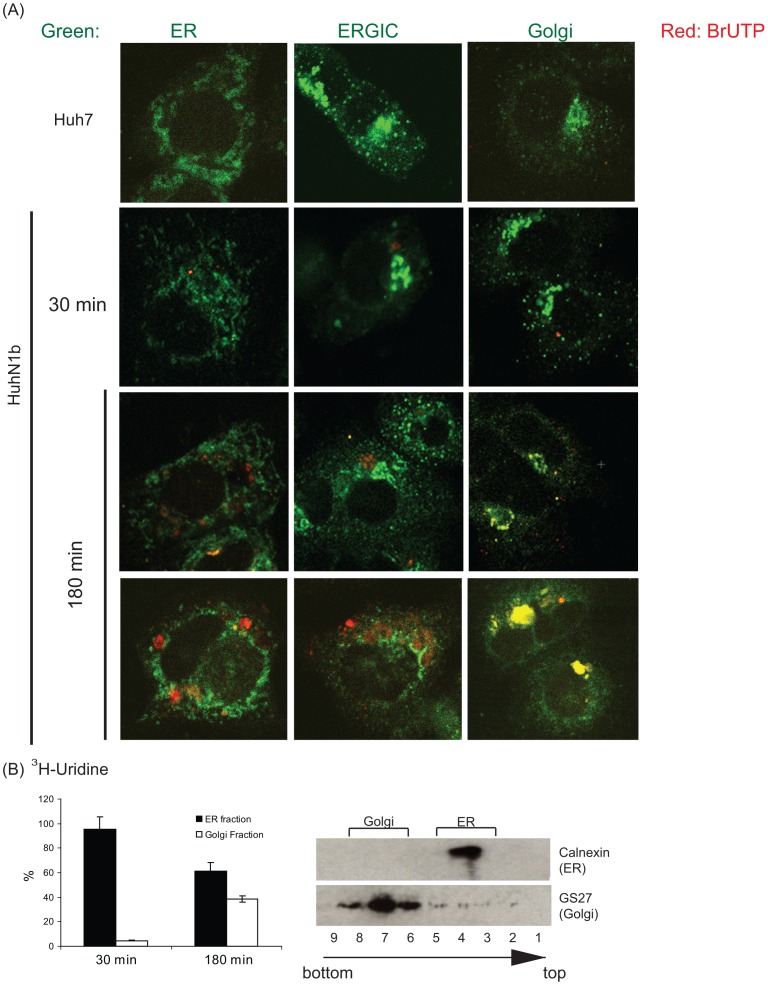
The translocation of newly-synthesized HCV RNA. HCV replicon cells were labeled with BrUTP (A) or ^3^H-Uridine (B) in the presence of actinomycin D and chased for up to 180 minutes. (A) Immunofluorescence staining with anti-BrdU and other organelle antibodies shows the colocalization of BrU-labeled HCV RNA with ER initially (30 min) and then with Golgi (180 min). (B) Fractionation of ER and Golgi by sucrose gradient. Fraction numbers and their gradient positions are noted at the bottom. ^3^H-Uridine-labeled RNA in the ER (fraction 4) and the Golgi (fraction 6–8) fractions were collected, and the radioactivity of ^3^H-Uridine-labeled RNA was counted. Immunoblotting of ER and Golgi makers demonstrates the separation of ER and Golgi by sucrose gradient fractionation.

In this study, we observed that HCV RNA exit from the site of RNA synthesis to the Golgi complex, a process that can be blocked by nocodazole, an inhibitor of the ER-Golgi transport pathway. Surprisingly, HCV protein translation was enhanced when HCV RNA movement was blocked, suggesting that the translation of viral proteins occurred near the site of RNA synthesis. We also found that the translation of HCV proteins was dependent on active RNA synthesis: inhibition of RNA synthesis resulted in decreased HCV viral protein synthesis before there was significant decrease in the total amount of HCV RNA, and that the replication-defective HCV RNA could not be translated efficiently *in vivo*. Finally, we found that at least most of the newly synthesized HCV proteins colocalized with the newly synthesized viral proteins. These findings together thus indicate that HCV replication and translation are coupled, in the sense that replication of viral RNA is linked to translation of viral RNA in situ.

## Materials and Methods

### Cell Lines, HCV Full-length and Subgenomic Constructs

Huh7 or Huh7.5 cells were grown at 37°C in Dulbecco’s modified Eagle medium (DMEM) supplemented with 10% fetal bovine serum (FBS) and nonessential amino acids. Huh7 cells were obtained from Dr. Sato's lab [20], and Huh7.5 cells were obtained from Dr. Rice’ lab [21]. Bicistronic HCV-N1b replicon was derived from the HCV-N strain with a neomycin-phosphotransferase (NPT) gene for selection as described [22]. Huh-Noe cells are stable cells derived from Huh7 cells with NPT expression as described previously [23], [24]. Huh-N1b replicon cell line and Huh-Neo cells containing an NPT gene were grown under the same conditions as Huh7 cells using the same media containing 0.5 mg/ml G418.

**Figure 2:**
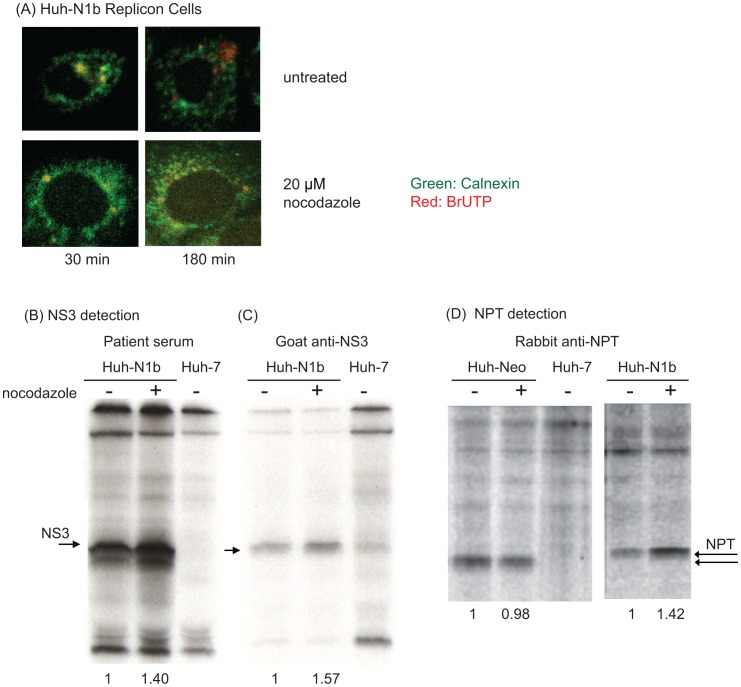
Increase in replicon RNA translation in nocodazole-treated HCV replicon cells. Huh-N1b cells was pre-treated with 20 µM nocodazole for 4 hours and then labeled with BrUTP or with ^35^S-Methionine. (A) The BrU-labeled RNA remained colocalized with calnexin (an ER amrker) even after 180 min. (B–D) Proteins were immunoprecipitated with (B) HCV patient serum, (C) Goat anti-NS3 antibody, or (D) Rabbit anti-NPT antibody. The immunoprecipitated products were detected by autoradiography. The nocodazole-pretreated Huh-N1b cells showed about 50% increase in NS3 and NPT translated from replicon RNA, whereas NPT translation in the Huh-Neo control cells was not affected by the nocodazole treatment.

### *In vitro* Transcription and Electroporation of HCV Full-length and Subgenomic RNA

HCV JFH1 and JFH-GND constructs were obtained from Dr. Wakita’s lab (NIID, Japan) [25]. Bicistronic replicon with either firefly luciferase (FFLuc) or Renilla luciferase (RLuc) gene was derived from HCV1bneo [22] by replacing NPT with either FFLuc or RLuc reporter gene. To prepare the template for *in vitro* transcription, the plasmids were digested by Xba I and Mungbean nuclease and gel-purified. For electroporation, Huh7 or Huh7.5 cells were trypsinized, washed and resuspended in serum-free DMEM. HCV replicon RNA or JFH full-length RNA were transcribed *in vitro* by T7 MegaScript (Ambion). A total of 6 to 10 µg of RNA and 10^7^ Huh7 cells were mixed and incubated on ice for 5 minutes and subjected to an electric pulse at 975 µF and 220 V. Cells were immediately transferred to 8 ml of DMEM containing 10% FBS for incubation.

**Figure 3:**
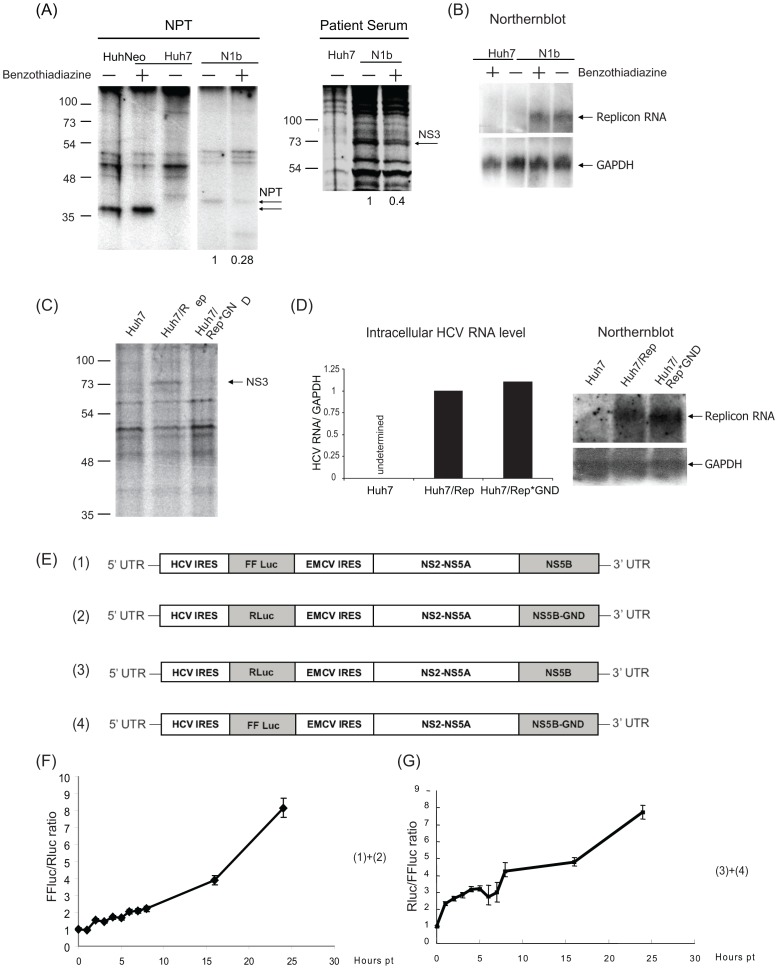
HCV RNA translation is dependent on the RNA transcription. (A), Mock- or Benzothiadiazine-treated Huh-N1b and Huh-Neo cells were labeled by ^35^S-Methionine for 4 hours, and followed by immunoprecipitation with anti-NPT or anti-NS3 antibodies or sera from hepatitis C patients. The immunoprecipitates were separated by SDS-PAGE and detected by autoradiography. (B) The intracellular replicon RNA was detected by Northern blotting. (C) Huh7 cells transfected with *in vitro* transcribed Rep or the replication-defective Rep*GND RNA were metabolically labeled with ^35^S-Methionine for 14 hours and followed by immunoprecipitation with anti-NS3 antibody. (D) The amounts of the intracellular HCV RNA in panel (C) were determined by realtime RT-PCR and Nothern blotting. The relative ratios of the HCV RNA/GAPDH mRNA in the different cells are presented. E) Structures of the bi-cistronic replicon reporter constructs used. Time course studies of the luciferase activity in cells transfected with constructs 1 and 2 (panel (F)) and constructs 3 and 4 (panel (G)) were measured by dual luciferase assay at various time points after transfection. The ratios of the FFluc/Rluc (F) or Rluc/FFluc (G) are presented. Error bars represent +/− standard deviation.

### Labeling and Immunofluorescene Staining of De Novo-synthesized Viral RNA and Newly-translated Peptides

Labeling of de novo-synthesized viral RNA, immunofluorescence staining and confocal microscopy were modified from the previously described methods [26]. Briefly, Huh7, Huh7.5 or replicon cells were plated on 8-well chamber slides at density of 1×10^4^ cells per well. Two days after seeding, cells were incubated with actinomycin D (10 µg/ml) for 1 hour to inhibit cellular RNA synthesis, and in some experiments, also with 20 nM of hippuristanol [27] for 1 hour to inhibit eIF4A-dependent protein synthesis, which represents most of the host cell protein synthesis. For immunofluorescence detection of de novo synthesized viral RNA, 2 mM of bromouridine triphosphate (BrUTP) was subsequently transfected into cells at 4°C for 15 min using Fugene 6 transfection reagent according to the manufacturer’s instructions (Roche). For live-cell imaging of both RNA and proteins, Cy5-UTP and BODIPY-FL-Lys-tRNA were cotransfected to label nascent HCV RNA and peptides, respectively. For immunofluorescence staining of both nascent viral RNA and proteins, Transcend biotinyl-Lys-RNA (Promega) [28] and BrUTP were instead used. The transfected cells were washed with phosphate-buffered saline (PBS) twice and incubated at 37°C with DMEM supplemented with 10% FBS for different periods of time. After incubation, cells were washed twice with PBS and subsequently fixed with 4% formaldehyde for 1 hr at 4°C. For permeabilization, the cells were treated with 0.1% Triton X-100 in PBS supplemented with 1% FBS for 30 min at RT. Primary antibodies were diluted in PBS containing 1% bovine serum albumin (BSA) and incubated with cells for 1 hr at RT. After three washes in PBS, the cells were incubated with fluorescein-isothiocyanate (FITC)-conjugated or Rhodamine-conjugated secondary antibodies, or Texas-red-conjugated streptoavidin diluted at a 1∶100 with PBS containing 5% BSA for 1 hr at RT. Then the cells were washed three times in PBS and mounted in Vectashield (Vector Laboratories).

**Figure 4:**
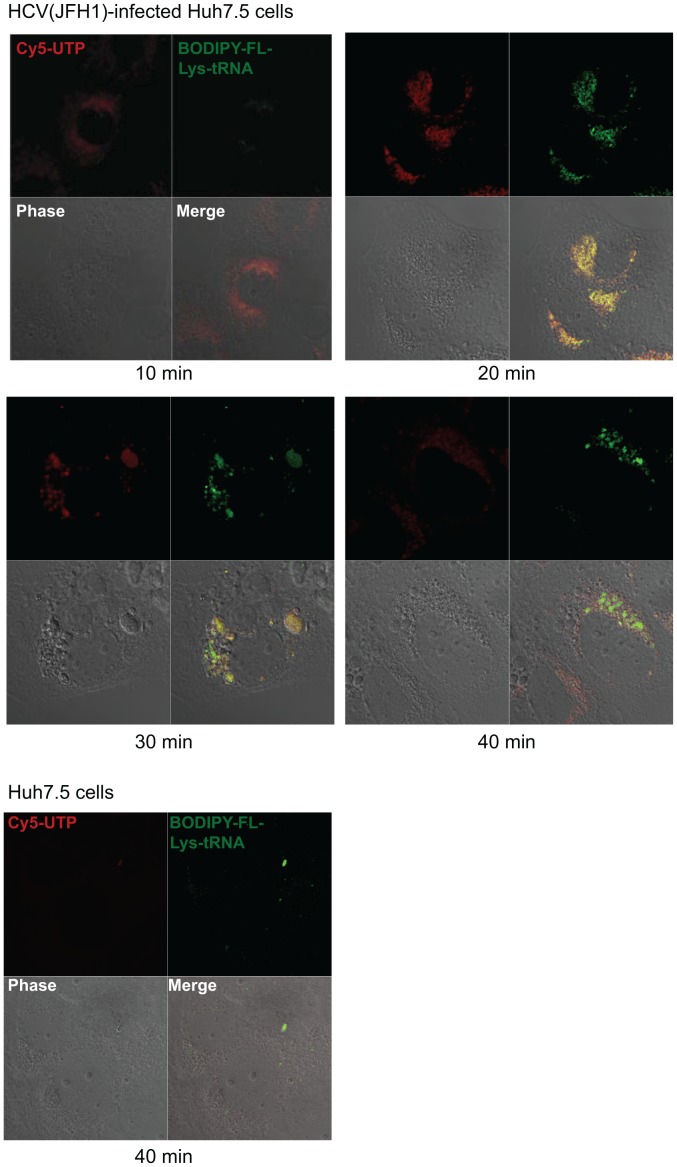
Double-labeling of newly-synthesized HCV RNA and newly synthesized viral peptides in JFH1-infected Huh-7.5 cells. Huh7.5 cells were infected with HCV JFH-1 strain for 2 days, and then were labeled with Cy5-UTP and BODIPY-FL-Lys-tRNA in the presence of actinomycin D and hippuristanol, which inhibit host RNA and protein synthesis, respectively. The cells were kept in 37°C chamber supplied with CO_2_ for live cell imaging on a Zeiss LSM 510 laser scanning confocal microscope. Images were taken after 10–40 minutes of chase. Newly-synthesized HCV RNA was the first to be detected (as shown in red) and was in a perinuclear pattern. Newly-translated HCV viral peptides (as shown in green) were detected at later time points, completely co-localized with the sites of RNA synthesis. No significant amount of Cy5-UTP and BODIPY-FL-Lys-tRNA labeling could be detected in naïve Huh7.5 cells (as a negative control) in the presence of actinomycin D and hippuristanol.

### Analysis of Intracellular Viral RNA by Northern Blotting and Real-time RT PCR

To determine the quantity of RNA by real-time PCR, a single-tube reaction was performed by using the TaqMan EZ RT-PCR Core Reagents (Applied Biosystems). Duplicate reactions for RNA standards and the samples were performed in 20-µl volume using 1 µl of HCV RNA, primers from HCV 5′ non-coding region (5′ GAG TGT CGT GCA GCC TCC A 3′ and 5′ CAC TCG CAA GCA CCC TAT CA 3′) of the HCV 1b sequence [29], and a fluorescent probe [5′ (FAM) CCC GCA AGA CTG CTA GCC GAG TAG TGT TGG (TAMRA) 3′] spanning these two regions. The RT step was performed at 60°C for 50 min, followed by 1 min at 50°C. The amplification condition was as follows: 95°C for 5 min and 50 cycles of denaturation at 94°C for 15 sec, annealing at 55°C for 10 sec, and extension at 69°C for 1 min.

**Figure 5:**
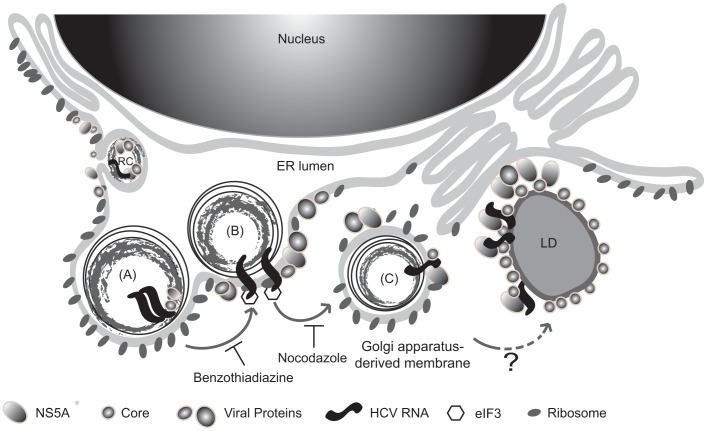
The proposed model of coupled replication/translation of HCV RNA. A proposed model of HCV replication-translation complex “replicasome”. As reported, HCV replication complexes are assembled at ER, and then bud into the ER lumen. Consequently, (A) HCV RNA replication is first initiated in the multi-layered vesicle structure derived from the ER membrane. (B) The newly synthesized HCV RNA is translated around the ER-derived vesicle [39], where there are membrane-associated ribosomes. Benzothiadiazine blocks HCV RNA transcription and therefore decreases translation. (C) The newly-synthesized HCV RNA is later transported away from ER; nocodazole inhibits this transportation. HCV RNA is then transported to Golgi-derived membrane and/or then the lipid droplet (LD) for packaging and assembly of virus particles.

Using the ABI Prism 7900 program, standard curves of the assays were obtained automatically by plotting the three hold values against each standard dilution of known concentration (10^1^–10^6^ copies per reaction) of HCV genotype 1b transcript. The same software was used to calculate the coefficients of regression. Values were normalized to that of GAPDH (Applied Biosystems). Each test was done in triplicate and averages were obtained.

### Fractionation of ER and Golgi Membrane

The procedure was based on the published method [30]. Cell lysates were applied to a discontinuous sucrose gradient composed of layers of 2 M, 1.3 M, 1.0 M and 0.6 M sucrose. The ER fraction was concentrated at the interface between 0.6 M and 1.0 M sucrose, and the Golgi fraction was concentrated at the interface between 2 M and 1.3 M. To determine the signal of ^3^H-Uridine-labeled RNA, the fractions were passed through DE81 membranes to concentrate the labeled RNA. The membranes were then counted by scintillation counter, and the ratio of signals from the ER and Golgi fractions were calculated.

## Results

### The Newly-synthesized HCV RNA Localizes to the ER and Moves Along with Anterograde Vesicle Trafficking

To visualize the replication of HCV RNA, we performed pulse BrUTP labeling in the actinomycin D-treated Huh-N1b replicon cells; under such conditions, only the viral RNA, which depends on RNA-dependent RNA polymerase, is labeled. After 15-minute labeling, the labeled RNA was chased in non-labeled media for 30 minutes to 3 hours, and the subcellular localization of BrU-labeled RNA was detected with anti-BrdU antibody and co-stained with individual organelle markers for ER (Calnexin), ERGIC (ERGIC53), and Golgi apparatus (GS27), respectively (Fig. 1A). Consistent with our previous report, the newly synthesized HCV RNA was initially colocalized with the ER marker (Fig. 1A). However, after 3 hours of chase, the majority of the labeled HCV RNA did not colocalize with the ER marker, but colocalized with the Golgi marker instead (Fig. 1A, bottom panels). We did not observe any significant colocalization between BrU-labeled HCV RNA and ERGIC53. As a control, the BrUTP signals could not be detected in the actinomycin D-treated Huh7 cells, while the immunofluorescence-staining patterns of the ER, ERGIC, and Golgi apparatus appeared similar between Huh7 and Huh-N1b cells (Fig. 1A).

The ER-to-Golgi trafficking of the newly synthesized RNA was further confirmed by biochemical analysis. The actinomycin D-treated Huh-N1b cells were labeled with ^3^H-uridine for 30 minutes and chased for 30 minutes to 3 hours. The labeled cell lysates were separated into ER and Golgi fractions by ultracentrifugation. Immunoblotting studies showed that the ER and the Golgi apparatus were efficiently separated by this procedure (Fig. 1B, right panel). The relative ratio of ^3^H-uridine-labeled RNA in the Golgi and the ER significantly increased over time (Fig. 1B). The result suggested that the newly-synthesized HCV RNA was transported from the ER-derived to the Golgi apparatus-derived membranes.

### HCV RNA Translation is Increased when Anterograde Vesicle Trafficking is Blocked

The ER-to-Golgi apparatus trafficking is known as the anterograde vesicle trafficking pathway, and can be blocked by nocodazole, which depolymerized microtubules and disrupts Golgi apparatus [31], [32]. When BrUTP labeling was performed in the presence of nocodazole, BrU-labeled RNA colocalized with ER (calnexin) even after 3 hours of chase (Fig. 2A). Under these conditions, the morphology of cells was not altered by the treatment. These results suggest that the anterograde vesicle trafficking is involved in the transport of HCV RNA after its synthesis. We then further investigated if this transportation is required for certain steps of the HCV life cycle. Previously it has been shown that prolonged (more than 24 hours) nocodazole treatment inhibits HCV replication and viral production [33]; we thus tested if the nocodazole treatment could affect HCV translation. Huh7 or Huh-N1b (HCV replicon) cells were pre-treated with nocodazole for 4 hours, and then labeled with ^35^S-Methionine for 4 hours to determine HCV translation activity (Fig. 2B). We determined the amounts of ^35^S-Methionine-labeled Neomycin-phospho-transferase (NPT) and HCV NS3 proteins, both of which are encoded from the HCV replicon RNA but under the control of separate IRES elements. We found that after a 4-hour nocodazole treatment, the total amount of the labeled NS3 protein, as detected by anti-NS3 or HCV patients’ sera, was significantly increased (Fig. 2, B and C). Similar observation was made for the NPT protein translated from the HCV replicon (Fig. 2D). Quantitation of the proteins showed a 40%–50% increase in both NPT and NS3 protein synthesis after a 4-hour nocodazole treatment. Since the translation of these two proteins was under the regulation of different sequence elements, these results suggest that the increase in HCV protein translation was not due to specific enhancement of HCV IRES-mediated translational activity. We also tested the effects of the nocodazole treatment on the NPT synthesis in the neomycin-resistant Huh-Neo cells, in which NPT is expressed from an integrated plasmid DNA. In contrast to HCV replicon cells, NPT synthesis in Huh-Neo cells was not affected by the nocodazole treatment (Fig. 2D,), indicating that the increase in HCV protein synthesis by the nocodazole treatment in HCV replicon cells was not due to enhancement of global translation.

These results are unexpected, raising a possibility that the newly-synthesized HCV RNA may be used for RNA translation in situ, without being transported away from the site of RNA synthesis. This result brought up an intriguing possibility that HCV RNA replication and translation are coupled and take place in the same replication complex.

### HCV RNA Translation is Dependent on the Transcriptional Activity of the RNA

To test the idea that the newly synthesized HCV RNA is used for translation in situ, we then investigated whether translation was dependent on active RNA synthesis. We used a specific NS5B polymerase inhibitor, Benzothiadiazine [34], [35], to inhibit HCV RNA synthesis and then examined the possible effects, if any, on HCV translation.

We first determined the efficiency and specificity of the inhibitor on ^3^H-uridine incorporation (Fig. S1A). Huh7-N1b replicon cells were pretreated with or without Benzothiadiazine for 16 hours and then with actinomycin D for an additional 1 hour prior to ^3^H-uridine labeling. Under this condition, ^3^H-uridine is expected to be incorporated into HCV RNA only, but not cellular RNA [30]. The data showed that, in Huh7 cells, actinomycin D almost completely inhibited uridine incorporation. However, in Huh-N1b cells, actinomycin D did not completely inhibit ^3^H-uridine incorporation; the residual incorporation likely represents HCV RNA synthesis, as confirmed by the autoradiography of the RNA products (Fig. S1A, lower panel). This residual RNA synthesis was inhibited by Benzothiadiazine. Furthermore, the Br-UTP label in Huh-N1b cells was detected as speckles in the perinuclear region; these speckles were not visible when the cells were treated with Benzothiadiazine (Fig. S1B). These results together indicate that Benzothiadiazine inhibits viral RNA synthesis specifically.

We also studied the effects of Benzothiadiazine on the steady-state level of replicon RNA by realtime RT-PCR analysis. The data showed that even after 16 hours of treatment, the total amount of replicon RNA in the cells was not significantly affected (Fig. S1C). Furthermore, the size of HCV RNA remained the same even after 16 hours of Benzothiadiazine treatment. After 2 days of treatment, however, the amounts of the replicon RNA decreased by about 50%. After 5 days, the RNA level dropped to 10% that of the control cells (Fig. S1D). As a comparison, nocodazole, which was reported to inhibit HCV RNA replication [33], had a smaller effect on the amounts of HCV RNA.

Having established the specificity of Benzothiadiazine on HCV RNA synthesis, we then labeled the Benzothiadiazine-treated cells with ^35^S-Met and immunoprecipitated HCV proteins from the cell extracts (Fig. 3A). At 4 hour post-treatment, the amounts of newly synthesized HCV NS3 and NPT proteins were significantly decreased after the Benzothiadiazine treatment (Fig. 3A), while the amount and size of HCV RNA were not significantly affected, as determined by realtime RT-PCR and Northern blot (Fig. 3B). In contrast, in Huh-Neo cells, the Benzothiadiazine treatment did not affect the translation of NPT (Fig. 3A), indicating that Benzothiadiazine specifically inhibited translation of HCV RNA.

We further used a replication-defective replicon RNA (GND mutation in the NS5B region) to assess if active RNA replication of HCV is required for HCV protein translation. A separate experiment using an *in vitro* translation system showed that both wild-type HCV replicon and its GND mutant RNAs could produce equivalent amounts of NS2 to NS5B proteins (data not shown), indicating that the open reading frames in these RNAs are intact. The wild-type HCV replicon (Rep) and its GND mutant (Rep*GND) RNAs were then transfected into Huh7 cells, and the transfected cells were metabolically labeled with^ 35^S-Met for 14 hours to detect protein syntheses by immunoprecipitation with anti-NS3 or -NPT antibodies. The result showed that, the GND mutant yielded very little NS3 as compared with the corresponding wildtype replicon RNA (Fig. 3C). The amounts of wildtype and mutant RNAs were equivalent at 14 hours post RNA electroporation (Fig. 3D). This result suggested that HCV RNA replication enhanced the efficiency of HCV RNA translation, but could not rule out the possibilities that this enhanced translation was due to quicker degradation of the Rep*GND mutant and/or a higher copy number of the Rep RNA as a result of RNA replication. To further investigate if replication-competent HCV replicon RNA was preferentially translated, we compared the translation activity of a bicistronic Firefly luciferase replicon RNA (Luc-Rep) and the comparable but replication-defective Renilla luciferase replicon GND mutant (RLuc-RepGND) (Fig. 3E). Both constructs were first tested by *in vitro* translation assay to ensure that the both reporters were functional (data not shown). Immediately after transfection into Huh7 cells, both luciferase activities were equivalent initially; however, the FFLuc/RLuc ratio increased over time (Fig. 3F). The reverse paired replicons, RLuc-Rep and Luc-GND, also gave a similar result (Fig. 3G). We observed 2–4 folds more luciferase activities translated from the replication-competent replicon RNA than those from the GND mutants at 8 hr post-transfection (Fig. 3F & G), at which time the incoming RNAs had not yet been degraded, indicating that the replication-competent HCV RNA was preferentially translated.

We further performed a similar study using the infectious HCV clone JFH1 and its replication-defective mutant, JFH1-GND, in a time-course study of HCV protein translation. Huh7 cells were transfected with JFH1 or JFH1/GND RNA, labeled with ^35^S-Met during the 0–8, 8–16, or 16–24 hours post-transfection, and followed by immunoprecipitation. The amounts of these two RNAs at 24 hours post-transfection were almost the same (Fig. S2B). However, only the infectious JFH RNA, but not its GND mutant, yielded detectable amounts of NS3 and NS5A at 16–24 hours posttransfection (Fig. S2A). The kinetics of HCV protein synthesis corresponded well with the previously reported kinetics of HCV RNA synthesis following HCV (JFH1) RNA transfection [36]. The result further supported the conclusion that the replication of HCV RNA is required for competent HCV RNA translation.

We next assessed if the replication and translation of HCV RNA occurred in the same subcellular localizations. Huh7.5 cells were infected with HCV (JFH1); at 2 days after infection, the cells were labeled with Cy5-UTP and BODIPY-FL-lys-tRNA in the presence of hippuristanol and actinomycin D, which blocked eIF4A-dependent translation and host RNA transcription, respectively [27], [37]. Under such a condition, Cy5-UTP will label newly synthesized HCV RNA, while BODIPY-FL-lys-tRNA will label only the newly synthesized HCV proteins since HCV translational initiation does not require eIF4A, which is blocked by hippuristanol [38]. A control experiment showed that neither dyes labeled the uninfected Huh7.5 cells (Fig. 4, lower panel). JHF-1-infected Huh7.5 cells were labeled with Cy5-UTP and BODIPY-FL-lys-tRNA for 15 minutes and then chased in unlabeled media from 10 to 40 minutes. The Cy5-U label could be detected at the early time point (10 min.) in the perinuclear region; BODIPY-FL-Lys-tRNA-labeled peptides were detected sparingly at this time, but gradually increased in intensity at later time points (Fig. 4). The earlier detection of Cy5-U label than BODIPY-FL-lys-tRNA label may have been due to the more efficient incorporation and more sensitive detection of the former label. Strong BODIPY-FL-lys-tRNA labeling signals were detected after 30 minutes of chase. Significantly, all of the BODIPY-FL-lys-tRNA labeled peptide colocalized with Cy5-U-labeled RNA at all the time points studied (Fig. 4). These findings suggest that HCV protein translation occurs on the newly synthesized RNA, and thus is likely coupled with RNA synthesis. From all the results above, we conclude that HCV RNA replication activity is a prerequisite to the efficient translation of HCV RNA.

## Discussion

The mechanisms of replication and translation of HCV RNA have been extensively studied in the past few years. However, the exact subcellular localization of HCV RNA replication and translation is still unclear. Evidence has previously been presented that HCV RNA replication occurs on the detergent-resistant membrane (DRM) possibly derived from the ER [23], [39]. In this report, the newly synthesized RNA was shown to be transported by the anterograde vesicle transport pathway. The microtubule-dependent mobility of newly-synthesized HCV RNA or the replication complex has also been described elsewhere [40]. Our data in this study further showed that the nocodazole treatment inhibited the transportation of the newly-synthesized RNA from the ER-derived replication complex to Golgi but did not inhibit the initiation of HCV RNA replication, since BrUTP labeling of HCV RNA occurred normally in the presence of nocodazole (Fig. 2). Intuitively, the newly-synthesized HCV RNA is expected to be transported to the site of the cellular translation machinery, similar to the case for cellular mRNAs, which are synthesized in the nucleus and transported to the cytoplasmic translation machinery for protein synthesis. However, we instead found that the movement of the HCV RNA from the ER to Golgi was not required for HCV translation, suggesting that the newly synthesized HCV RNA is used for translation near the site of HCV RNA synthesis before being transported away. Furthermore, we showed that active RNA replication was a prerequisite for efficient HCV translation. This conclusion was demonstrated using four different approaches, including studying the effects of an HCV RNA polymerase inhibitor on HCV protein translation (Fig. 3A), comparing the translation efficiencies of wildtype and replication-defective replicons (Fig. 3C) and those of infectious and non replicating JFH strain of HCV (Fig. S2A), and also by determining the relative translation efficiencies of the replicating and non-replicating dual luciferase reporter plasmids (Figs. 3F–G). Finally, we showed that the newly synthesized viral proteins almost completely colocalized with the newly synthesized viral RNA, suggesting that the sites of HCV RNA replication and protein translation nearly overlap. This mechanism of coupled RNA replication and translation may explain the previous findings that many cellular proteins, such as PTB [9], [26], La antigen [10], [13] and SYNCRIP [11], [14], are involved in both the replication and translation in the HCV life cycle. The close proximity of these two machineries will allow for ready switches between translation and replication.

Although coupling of translation and RNA replication has been reported for many RNA viruses [16], [17], [41], [42]_ENREF_37, the HCV case appears to be unique. For example, translation and replication of poliovirus RNA are coupled, but in the sense that RNA transcription is dependent on viral translation in *ci*s [16]. Insertion of an early termination codon resulted in lower efficiency of poliovirus RNA replication. The translation and replication are regulated by the binding of different cellular or viral proteins to the 5? UTR of poliovirus RNA [43]–[45]. Also, the microtubule-dependent movement of poliovirus viral RNA is associated with the replication activity of viral RNA [46]. While the inactive replication complexes reside at microtubule-organizing center (MTOC), the replicating viral RNA is localized at the perinuclear sites [46]. Thus, the RNA movement is required for poliovirus replication, in contrast to the situation with HCV. In HCV, nocodazole did not inhibit viral RNA replication; also, the newly-synthesized HCV RNA failed to exit from ER after the nocodazole treatment and yet, protein translation increased; thus, the cytoskeleton-assisted movement of the newly-synthesized HCV RNA is not required for RNA translation. Thus, in HCV, the observed movement of the viral RNA from the ER-derived to the Golgi-derived membrane appears to be required for other steps of HCV replication, rather than protein translation. Due to the fact that the viral structural proteins are absent in the HCV replicon cells, this RNA movement is likely mediated by viral NS proteins, such as NS5A, which has been reported to target Golgi apparatus [47]. In a kinetics study examining the appearance of the newly synthesized HCV RNA and HCV proteins in the HCV (JFH-1)-infected cell, we also found that all of the newly synthesized proteins were at the site of newly synthesized RNA (Fig. 4). Thus, there appears to be a replication complex that carries out both replication and translation. This concept is novel to the known mechanisms of RNA virus translation and transcription.

These findings raised an important issue, namely, how the initial viral translation is carried out, since, as a positive-strand RNA virus, the very initial round of translation from the incoming HCV viral genome has to take place before viral RNA replication can occur. Conceivably, the free viral RNA genome generated by uncoating of the incoming virion in the endosome (or from the transfected viral RNA or replicons) can associate with ribosomes on the rough ER and be translated in an RNA replication-independent manner. Such translation is likely of low efficiency, but is sufficient to support first round of HCV RNA translation. These initial viral protein products and RNAs will then be encased into the membranous replication complex and become part of the replication-translation machinery. The latter process will then become the main mechanism of HCV replication-translation.

In summary, we propose the following pathway for HCV RNA replication and translation (Fig. 5). Previous studies have shown that HCV RNA replication takes place in an ER-derived membranous vesicle [39]. The newly-synthesized viral RNA will be translated immediately after being synthesized in or around the vesicle. Benzothiadiazine treatments inhibited HCV RNA transcription and therefore inhibited HCV RNA translation as well (Fig. 5, A–B). After translation has occurred, HCV RNA is then transported via anterograde vesicle trafficking pathway away from the ER to a membrane compartment associated with the Golgi apparatus (Fig. 5C). Several other reports suggest that HCV RNA may be transported along with viral proteins (core and NS5A) to lipid droplets, where viral assembly takes place [48]; however, the kinetics of this process is unclear. Further studies in dissecting HCV RNA transportation during HCV infection will be valuable for understanding HCV life cycle comprehensively. In conclusion, this replication and translation machinery constitutes the viral “replicasome”, which may reflect the membranous webs observed previously.

## Supporting Information

Figure S1 **Benzothiadiazine treatment in Huh-N1b replicon cells.** Huh7 and Huh-N1b cells pre-treated with actinomycin D, Benzothiadiazine (NS5B inhibitor), or combination of both for 4 hours, and then labeled with ^3^H-Uridine (A) or BrUTP (B). RNA synthesis was detected by RNA precipitation followed by scintillation counting (A), by immunofluorescence staining (B), or by real-time RT-PCR (C, D). Autoradiography of the H^3^-uridine-labeled RNA was also performed (bottom, panel A). The relative amounts of intracellular HCV RNA after the various treatments are shown in (C, D). Benzothiadiazine specifically inhibited viral transcription, but did not affect the intracellular replicon RNA levels under this condition (C). (D), long-term Benzothiadiazine treatment (over 2 days) significantly decreased the replicon RNA levels.
(TIF)

Figure S2 **Preferential translation of JFH1 wildtype over the GND mutant HCV RNA.** (A) A time-course study of HCV NS protein translation in cells transfected with JFH1 or its GND mutant. The cells were labeled with S^35^-methionine from 0–8, 8–16 and 16–24 hours posttransfection and followed by immunoprecipitation with anti-NS3 or HCV patient serum. NS3 and NS5A were detected by immunoprecipitation and separated by SDS-PAGE. (B) The corresponding intracellular HCV RNA levels in (A). The intracellular RNA levels at 24 hour post-transfection were determined by real-time RT-PCR.
(TIF)
