# Supplementary figures and images for: Hepatitis C Virus Translation Preferentially Depends on Active RNA Replication
Source: PLoS One. 2012 Aug 24;7(8):e43600. doi: 10.1371/journal.pone.0043600 (PMC3427374; doi:10.1371/journal.pone.0043600)

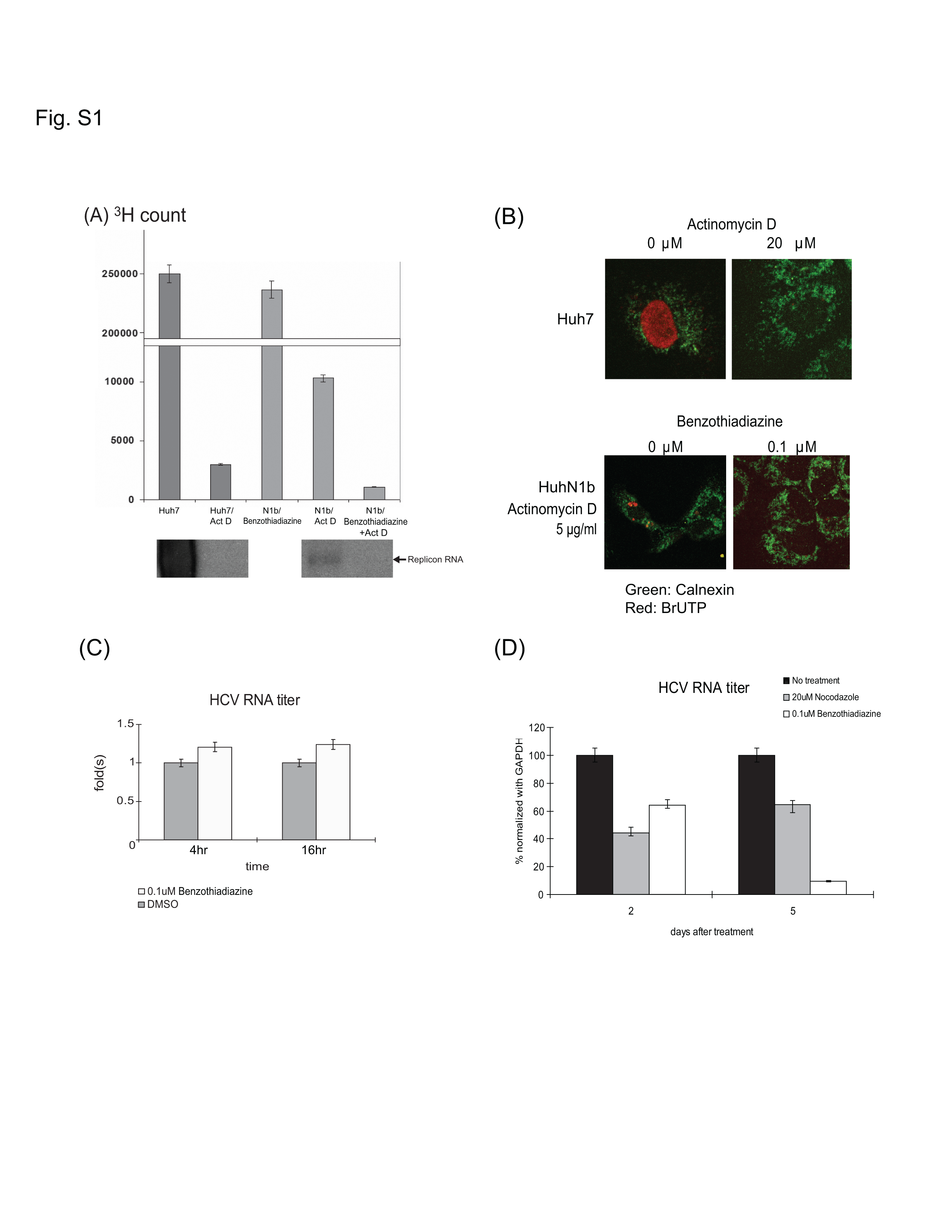

Supplement: Figure S1 — Benzothiadiazine treatment in Huh-N1b replicon cells. Huh7 and Huh-N1b cells pre-treated with actinomycin D, Benzothiadiazine (NS5B inhibitor), or combination of both for 4 hours, and then labeled with 3H-Uridine (A) or BrUTP (B). RNA synthesis was detected by RNA precipitation followed by scintillation counting (A), by immunofluorescence staining (B), or by real-time RT-PCR (C, D). Autoradiography of the H3-uridine-labeled RNA was also performed (bottom, panel A). The relative amounts of intracellular HCV RNA after the various treatments are shown in (C, D). Benzothiadiazine specifically inhibited viral transcription, but did not affect the intracellular replicon RNA levels under this condition (C). (D), long-term Benzothiadiazine treatment (over 2 days) significantly decreased the replicon RNA levels. (TIF) [file pone.0043600.s001.tif]

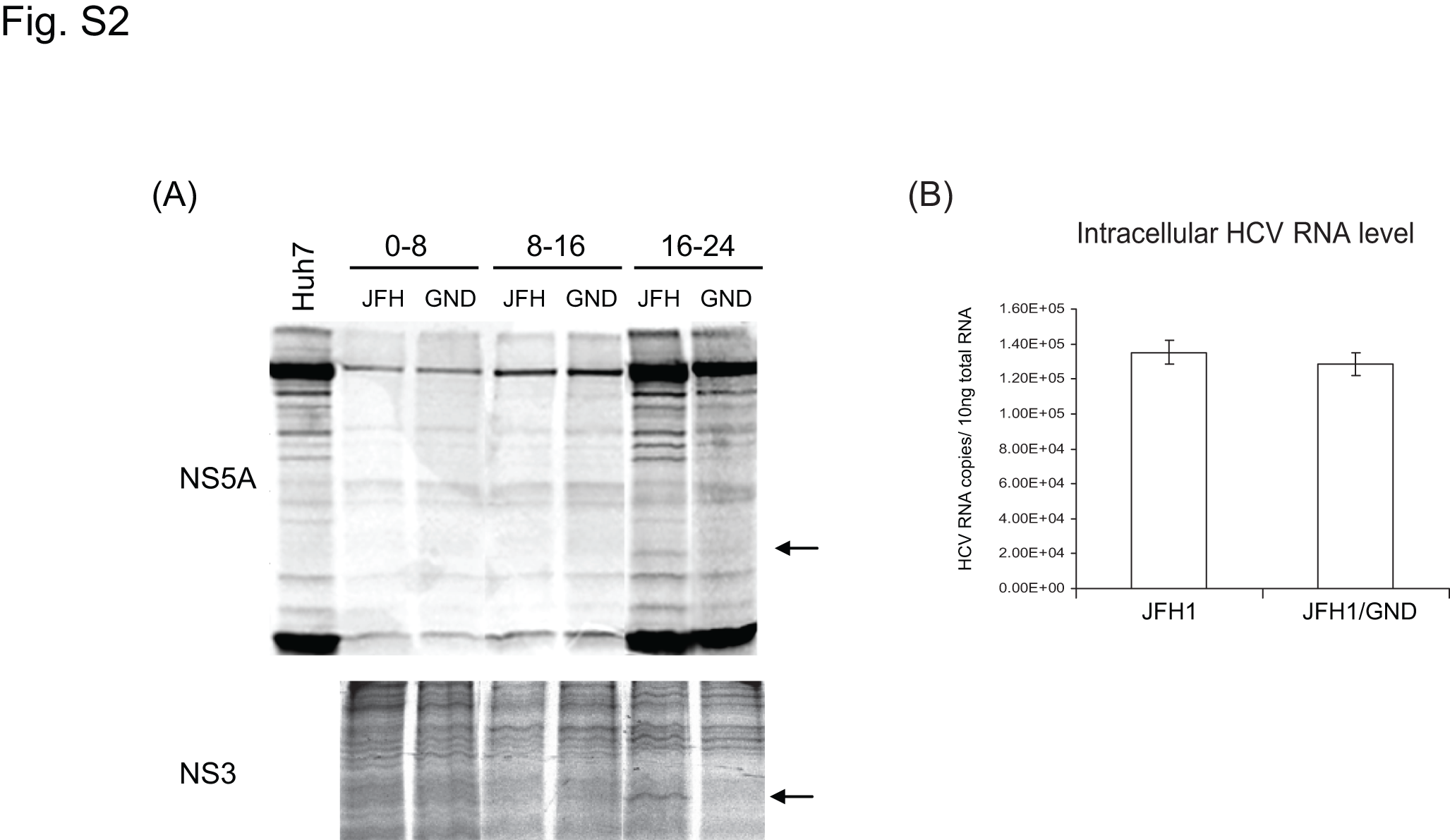

Supplement: Figure S2 — Preferential translation of JFH1 wildtype over the GND mutant HCV RNA. (A) A time-course study of HCV NS protein translation in cells transfected with JFH1 or its GND mutant. The cells were labeled with S35-methionine from 0–8, 8–16 and 16–24 hours posttransfection and followed by immunoprecipitation with anti-NS3 or HCV patient serum. NS3 and NS5A were detected by immunoprecipitation and separated by SDS-PAGE. (B) The corresponding intracellular HCV RNA levels in (A). The intracellular RNA levels at 24 hour post-transfection were determined by real-time RT-PCR. (TIF) [file pone.0043600.s002.tif]
